# Paediatric haemodynamic modelling: development and experimental validation using quantitative flow MRI

**DOI:** 10.1186/s41747-020-0146-x

**Published:** 2020-03-16

**Authors:** Parvin Mohammadyari, Giacomo Gadda, Angelo Taibi, Josep Munuera del Cerro

**Affiliations:** 1 Department of Physics and Earth Sciences, University of Ferrara, Via Giuseppe Saragat, 1, 44122 Ferrara, Italy; 2 National Institute for Nuclear Physics (INFN) - Section of Ferrara, Via Giuseppe Saragat, 1, 44122 Ferrara, Italy; 3 Department of Diagnostic Imaging, Fundació Sant Joan de Déu, Passeig de Sant Joan de Déu, 2, 08950 Esplugues de Llobregat, Barcelona, Spain

**Keywords:** Blood pressure, Cardiovascular system, Computer simulation, Hemodynamics, Pediatrics

## Abstract

**Background:**

Congenital vascular disease is one of the leading causes of death in paediatric age. Despite the importance of paediatric haemodynamics, large investigations have been devoted to the evaluation of circulation in adults. The novelty of this study consists in the development of a well calibrated mathematical model of cardiovascular circulation in paediatric subjects. To reach the purpose, a model for adult circulation was modified and recalibrated with experimental data and literature from children to be able to calculate the flow rates and pressures in the brain and neck.

**Methods:**

The haemodynamic model simulates the 76 main arteries, together with the main veins in brain and neck. A proper magnetic resonance imaging (MRI) dataset of 29 volunteers aged 12 ± 5 years (mean ± standard deviation) was used to extract age-dependent physiological and clinical parameters such as heart rate, flow rate, vessel cross section area, and blood pressure. The computational model was calibrated using such experimental data. The paediatric and adult model results were compared.

**Results:**

Increase of the vessels stiffness due to aging contributes to a flow rate decrease while blood pressure increases. In accordance, our simulation results show about 16% decrease in mean pressure of internal jugular vein in paediatric rather than adult subjects. The model outcomes indicated about 88% correlation with MRI data.

**Conclusions:**

The mathematical model simulates the paediatric head and neck blood circulation. The model provides detailed information of human haemodynamics including arterial and venous network to study both paediatric and adult blood circulation.

## Key points

- Aging affects the physiology of the cardiovascular system.
- Age-related physiological parameters need to be considered in a paediatric haemodynamic model.
- Our computer simulation validated by MRI at the level of head and neck provides detailed information to study haemodynamics in both paediatric and adult population.

## Background

Cardiovascular disorders are one of the most common forms of congenital defects in new-borns. The number of involved paediatric patients is lower than that of acquired cardiovascular disorders in adults; however, the mortality rate is higher [1]. Therefore, the importance of studying the paediatric vascular system is straightforward. Also, it is proven that some physiological parameters such as cardiac output (CO), cerebral blood flow (CBF), and arterial stiffness are age-dependent [2, 3]. The importance is emphasised by the claim: “children are not small adults” [4]. As a consequence, it is inappropriate to generalise or extrapolate data from adult to calculate the parameters and build a paediatric model [4, 5].

The cardiovascular system is a challenging subject of study due to the complex geometry and the high number of enrolled physiological parameters [6]. In the case of paediatric studies, the difficulty is higher because of the substantial limitations such as lack of standardising data due to patient intolerance to the clinical examinations, different physiology, and higher tissue sensitivity in comparison with adults. These barriers limit the options for choosing proper diagnostic methods [5]. Thereby, the need for more paediatric cardiovascular studies is frequently highlighted [2, 3, 5].

The physiological parameters undergo a progressive change with age. However, by the age of 8−12 years, the body reaches a relatively mature stage that resembles the adult body [7]. For instance, the CBF at rest increases during infancy until early childhood and then decreases about 40 to 50% until adulthood [2, 5, 8]. For this reason, it is possible to use the anatomical map and mathematical equations of the adult model as a basement in order to establish a paediatric model. However, the physiological age-dependent parameters such as vessels resistance and capacitance should be adjusted and calibrated for such paediatric age to regulate the blood flow and pressure calculated by the model. By this, we are allowed to use the same equations for the adult model with recalculated physiological parameters.

Mathematical modeling and computer simulation have been developing rapidly to study blood circulation [9]. Their benefits are not only limited to the understanding of the fluid behaviour in vessels but also for surgical planning and intervention [1]. Most of the numerical studies were dedicated to computational fluid dynamics investigation in modeling the cardiovascular and pulmonary vessel diseases while less attention has been paid to the study of the neck and brain vascular diseases [6, 10]. Models are regulated on the basis of physics and mechanics rules to understand the influences of the involved biological factors [11–13]. Moreover, pathological cases can be simulated starting from a model previously calibrated to mimic healthy subjects [14]. Indeed, such modeling must carry on comprehensive information about the morphology of the human vessels tree that is crucial to understand how a given change of the physiological parameters can affect the results. That is our motivation to improve the anatomic map of the model.

Anatomically speaking, the internal jugular veins (IJVs) connect the superior vena cava to the venous sinus in the skull. Each of the two IJVs is conventionally subdivided into three segments (J3, J2, and J1, from the head to the heart) and its cross-sectional area increases along the same direction [15, 16]. Due to their large cross-sectional area, IJVs are the main blood outflow pathway from the brain in the supine position [17].

Vertebral veins, internal venous plexus, and deep cervical veins are alternative pathways [13, 18]. There are communicating pathways between external jugular veins (which collect blood from temporal branches [15, 16]), IJVs, and deep cervical veins, playing an important role in improving the accuracy of the model output [18].

In terms of clinical needs, a large group of diseases which are known as different types of stenosis (like Moyamoya disease [19] and Chiari malformation [20]) can be studied by using a comprehensive mathematical model. Therefore, computational modeling can be considered as a powerful tool for clinical applications.

In this study, the mathematical model built by Gadda et al. [11, 14, 21] is modified to investigate more precisely the human brain drainage. The model considers the cardiovascular system as an electrical circuit where blood flow rate and pressure at the *x* anatomical level represent the electrical current and voltage, respectively. Accordingly, physiologic parameters of blood circulation are calculated [13, 14].

Few literature explored the internal and external cranial haemodynamics through mathematical models of the whole body circulation, and none of them relates to paediatrics. The aim of this study was to introduce a haemodynamic computational model for paediatrics. To do that, we managed a magnetic resonance imaging (MRI) dataset from normal subjects. Physiological age-related parameters were calculated by both the dataset and literature to adjust CO, CBF, and respiratory rate for paediatrics.

## Methods

### Patients

Patient selection and MRI acquisition took place at the Sant Joan de Déu Hospital (Barcelona, Spain). Twenty-nine healthy volunteers participated in the study (Table 1). Subjects were chosen among all the patients for whom a brain MRI had been requested. We considered only subjects with recognised normal neck and brain blood circulation that signed the informed consent to participate as a volunteer. The study was approved by the ethical committee of the hospital.

**Table 1:** Characteristic of the enrolled subjects

Demographic characteristics	
Total number of subjects	29
Sex (females:males)	14:15
Age (years)	12 ± 5
Age range (years)	2−18
Weight (kg)	43 ± 15
Systolic blood pressure (mmHg)	114 ± 9
Heart rate (beats per min)	83 ± 16
Clinical characteristics	
Headache	10
Epilepsy	4
Cyst	2
Hearing loss	2
Down syndrome	1
Acondroplasia	1
Diplopia	1
Tremor	1
Vertigo	1
Encephalopathy (NBIA_PKAN mutation)	1
Unknown encephalopathy-genetic origin	2
Cavernoma	1
Psychosis	1
Control trauma	1

Data are given as absolute frequencies or mean ± standard deviation

### MRI protocol

The volunteers were imaged using a 3-T scanner (Ingenia, Philips, Amsterdam, Netherlands) with a 32-channel head coil. Phase-contrast sequences were used to collect two-dimensional quantitative flow images of the neck at the vertebral level C2/C3 (J3), C5/C6 (J2), and at the brain level including the Willis circle and Sylvian aqueduct, with the subject in the supine position. The common technical parameters of the sequences were as follows: repetition time 9.1 ms; echo time 5.4 ms; flip angle 15°; slice thickness 4 mm; field of view 150 mm× 150 mm; reconstruction voxel size 0.6 mm × 0.6 mm; in-plane spatial resolution 1 mm^3^; temporal resolution 15 frames per cardiac cycle. Acquisitions were synchronised with the electrocardiogram of the scanned volunteer. We used phase images to extract data such as flow rate and pressure, and magnitude images to check the anatomy and improve the anatomic map of the model.

Two ranges of velocity encoding were tested in three subjects to improve the image quality and avoid aliasing effects. After this test, we chose the velocity encoding of 60 and 120 cm/s for veins and arteries, respectively, and of 10 cm/s to study the Sylvian aqueduct. Seven sequences were performed for each subject (Table 2). Resulting images were named by vein or artery at a specified anatomic location (Additional file 1).

**Table 2:** Velocity encoding and acquisition time of the applied quantitative-flow two-dimensional phase-contrast sequences

Imaged structures	Velocity encoding (cm/s)	Acquisition time (s)
Sylvian aqueduct (cerebrospinal fluid)	10	97
Brain arteries	120	123
Brain veins	60	124
Arteries at vertebral level C2/C3	120	125
Veins at vertebral level C2/C3	60	153
Arteries at vertebral level C5/C6	120	125
Veins at vertebral level C5/C6	60	153

Regions of interest (ROIs) were drawn manually on all the vessels on each sequence and then cross-sectional area of the vessels, blood flow, velocity, delay time, and pressure gradients were extracted by the Philips software (Philips IntelliSpace Portal, version 10). Blood flow was calculated by multiplying the mean velocity by the cross-sectional area of the region of interest, while pressure gradient was calculated by using the modified Bernoulli equation $\Delta  P=4\Big({V}_p^2$), where *V*_*p*_ is the peak velocity of blood [22].

After drawing the regions of interest on all the 15 frames acquired at each anatomical plane, the software calculated all the parameters. If the signal intensity was low (mostly in the temporal veins), the software did not calculate the pressure gradient. In other words, we eliminated from the study all the vessels with flow rate less than 0.05 mL/s because of the low signal-to-noise ratio. Otherwise, resolution was enough to include the acquired data in the dataset.

### Mathematical modeling

The model consists of a 0-D and 1-D algorithm [11]. In the 0-D algorithm, the intracranial autoregulation mechanisms and cerebral outflow were modeled according to the model by Ursino et al. [12, 23]. Each vessel was considered as an electric element with a given value of resistance, capacitance and conductance (Additional file 1) [14, 21, 23–27]. Equations satisfy the momentum and mass conservation laws and describe the pressure-area relationship. The model is managed by the software package MATLAB-Simulink 2017b.

### Adjusting the physiologic parameters

Physiology of paediatric cardiovascular system is different from the adult and wrongly based on studies involving adults [5]. Thus, the adult model needs appropriate modifications. Heart rate (HR), systolic blood pressure (SBP), and diastolic blood pressure (DBP) in paediatric subjects are different than in adults [4]. The mean age of the volunteers enrolled in our dataset was 12 ± 5 years, while the measured average HR was 83 ± 16 beats per min (mean ± standard deviation [SD]). Therefore, we set the duration of one cardiac cycle to 0.7 s (against a duration of 0.8 s in the adult model). The mean value of SBP (in mmHg) was calculated by using the equation SBP = 90 + 2(*Age*), where age is measured in years [28]. Therefore, the SBP for our dataset was equal to 114 ± 9 mmHg (mean ± SD).

**Fig. 1:**
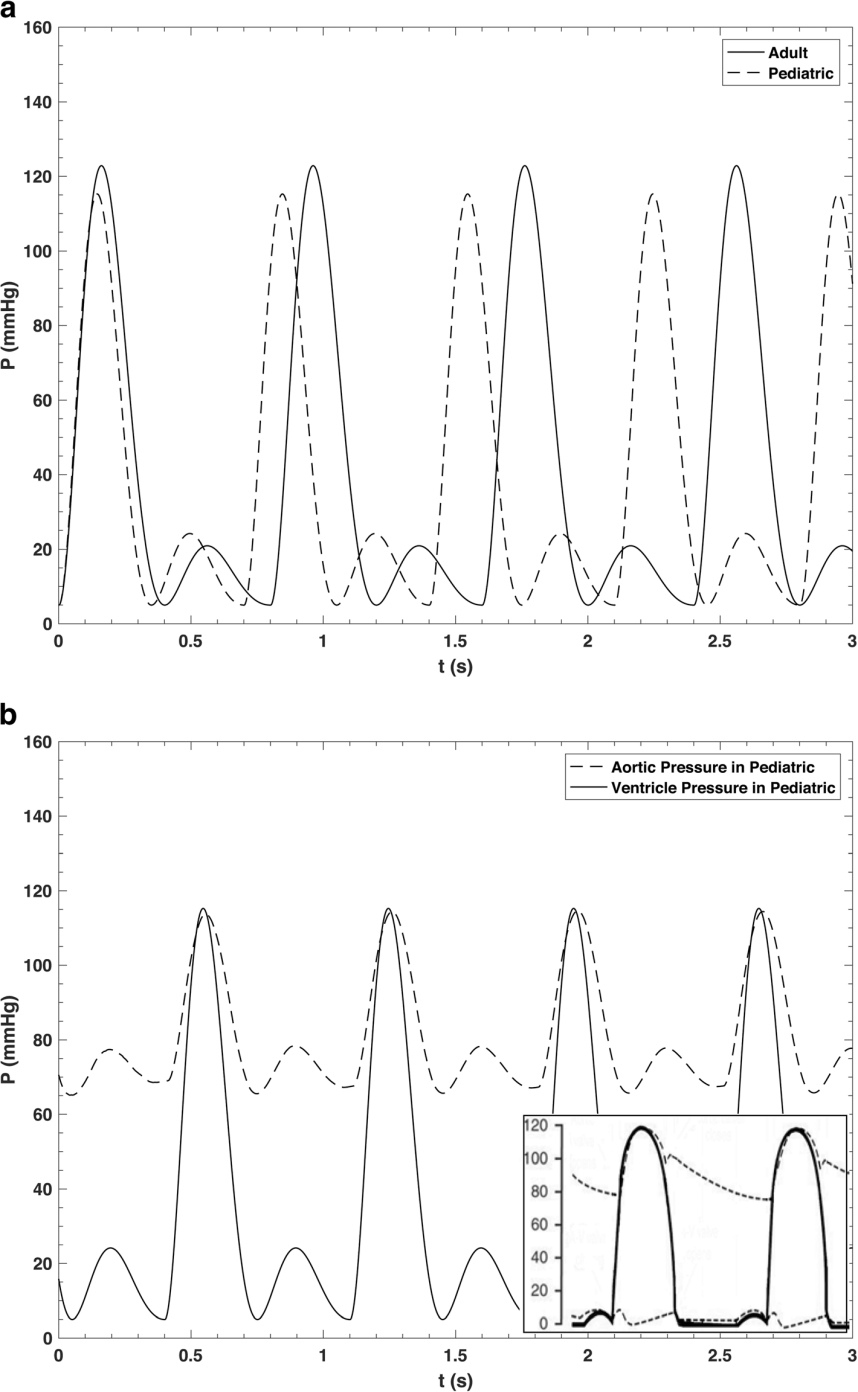
The simulated cardiac pressure waveform. **a** Comparison of the aortic and ventricle pressure wave in paediatric. **b** Comparison of the ventricle pressure wave in paediatric and adult. The inset shows the physiological pattern

For what concern the mean value of DBP, we referred to literature [28–30]. From the literature we know that the respiratory rate in adults and old-children are about 15 and 20 breaths/min, respectively [30]. We tuned the respiratory rate in the model accordingly. By setting the model with these new parameters, the new simulated cardiac pulse waves at the level of aorta and ventricle were representative of a paediatric cohort. In Fig. 1, such pulse waves are presented and compared with the results of the adult setting. The generated pulse wave is compared with the physiologic reference pattern for adults [24] represented in the inset of Fig. 1. The parameters were also regulated to generate the pressure waveform at the level of ascending aorta to satisfy the SBP and DBP according to the mean values from our database and the paediatric cardiopulmonary care guide [27]. More modifications were not necessary because there is no significant correlation between the mean value of systolic to diastolic ratio and age or body surface [31].

Other relevant age-related parameters to regulate the pressure and flow rate in the 1-D part of the model are total blood volume (TBV), cerebral blood volume (CBV), and cardiac volume output ratio index (CBV/CO) [2]. The CO is calculated by multiplying HR by the difference between end-diastolic volume and end-systolic volume [17]. Since the ascending aorta was not in the selected region of our study, we were not able to calculate the cardiac volume output ratio index. This value was taken from literature. Its mean value in infants, paediatric subjects with the same mean age of our dataset, and adults is 34.0%, 22.9%, and 14.3%, respectively [2, 32]. The simulated flow ratio percentage of CBF to the ascending aorta flow was tuned to be about 22.9%. According to literature, the paediatric TBV and CBF have the mean value of 3.4 ± 1.2 L (mean ± SD) and 1101 ± 258 mL/min (mean ± SD) (80 mL blood/100 mL brain), respectively [5, 32]. The weight of the brain is reported as 1100.7 ± 209.6 g (mean ± SD) and 1189 ± 99 g (mean ± SD) in paediatric subjects and adults, respectively [2, 32]. For adults, the reported mean TBV and CBF are 5.6 ± 0.3 L and 750 (490−770) mL/min (mean and min to max range) (70 mL blood/100 mL of brain), respectively [2, 11, 13, 32].

### Adjusting the vessels anatomy map of the model

Peripheral vessels play a considerable role in the brain and head drainage. In cases of obstructions of the main pathways, alternative routes compensate the blood outflow [13, 16, 17]. We measured the flow rate at different vessels (vessels with flow rate less than 0.05 mL/s were eliminated from the study because of the low signal-to-noise ratio). We adjusted the anatomic map of the model to improve its ability to evaluate both physiological and pathological cases. The full list of vessels added in this work is reported in Table 3.

**Table 3:** Vessels added in the present adjustment of the mathematical model

Veins	
External jugular (right and left)	
Deep cervical (right and left)	
External-internal jugular connection (right and left)	
Internal jugular-deep cervical connection (right and left)	
Arteries	
Internal carotid (right and left)	
Middle cerebral (right and left)	
Anterior cerebral (right and left)	
Posterior cerebral (right and left)	
Basilar	
Anterior communicating	
Posterior communicating (right and left)	
External carotid (right and left)	
Ophthalmic (right and left)	
Facial (right and left)	

**Fig. 2:**
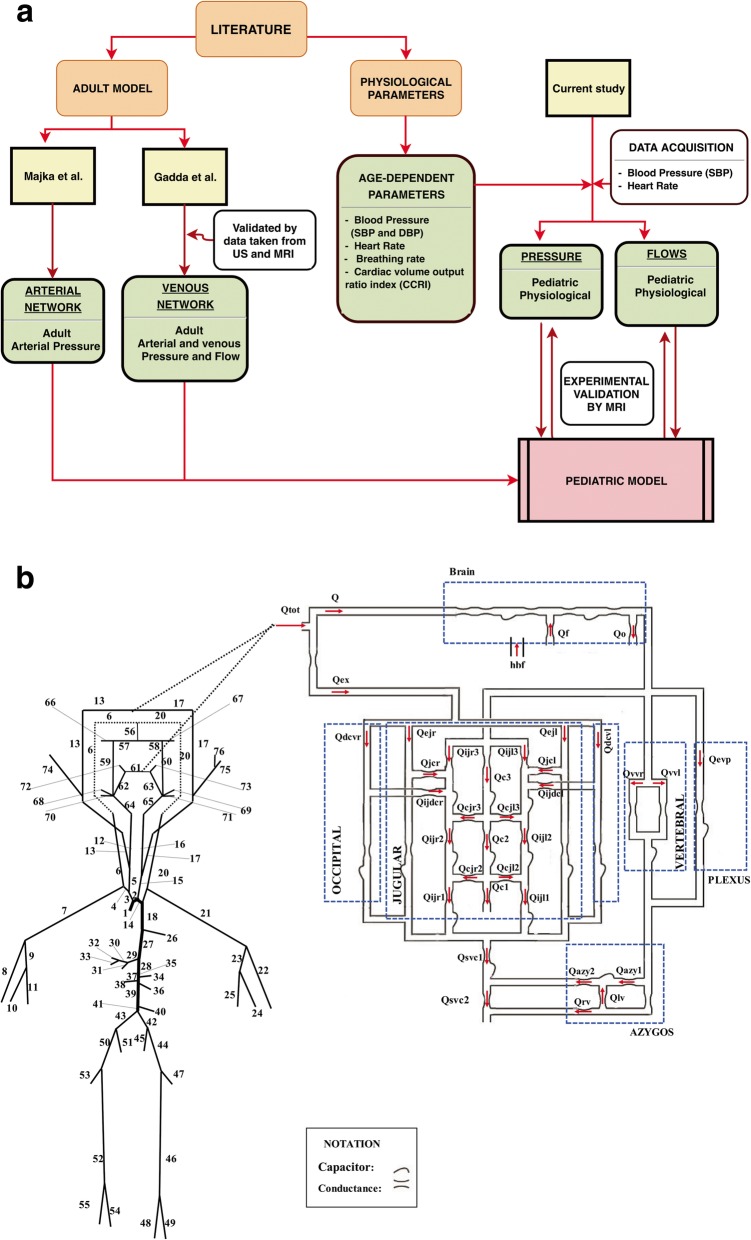
**a** Flowchart of all the contributions used to build the paediatric model. **b** Scheme of the modified haemodynamic model with all the new anatomical adjustments. *hbf* Cerebrospinal fluid possibly injected into or subtracted from the cranial cavity, *azy1* Distal azygos, *azy2* Proximal azygos, *c1* Lower segment of the collateral network, *c2* Middle segment of the collateral network, *c3* Upper segment of the collateral network, *cjl2* Lower anastomotic connection (left side), *cjl3* Upper anastomotic connection (left side), *cjr2* Upper anastomotic connection (right side), *cjr3* Upper anastomotic connection (right side), *dcvl* Left deep cervical vein, *dcvr* Right deep cervical vein, *ejl* Left external jugular vein, *ejr* Right external jugular vein, *evp* External venous plexus, *ex* Extracerebral, *Q*_*f*_ Cerebrospinal fluid formation rate, *ijdcl* Internal jugular-deep cervical connection (left side), *ijdcr* Internal jugular-deep cervical connection (right side), *ijl1* Lower segment of the left internal jugular vein, *ijl2* Middle segment of the left internal jugular vein, *ijl3* Upper segment of the left internal jugular vein, *ijr1* Lower segment of the right internal jugular vein, *ijr2* Middle segment of the right internal jugular vein, *ijr3* Upper segment of the right internal jugular vein, *jcl* External-internal jugular connection (left side), *jcr* External-internal jugular connection (right side), *lv* Lumbar vein, *Q*_*o*_ Cerebrospinal fluid outflow rate, *rv* Renal vein, *svc1* Superior vena cava (upper segment), *svc2* Superior vena cava (lower segment), *vvl* Left vertebral vein, *vvr* Right vertebral vein

The vascular structure already included in the model were the following: internal jugular veins, anastomotic connections, azygos plexus, cerebrospinal inflow, and outflow pathways (Fig. 2b) [11, 14, 21]; main arterial tree of the human body outside the braincase (Fig. 2b, arteries 1 to 55) [25, 26]

We worked in order to keep the proper relative magnitude among flow rates in the three segments of the IJV [14, 21, 33]. A summary of our work strategy is reported in the flowchart in Fig. 2a, which shows all the contributions used to build the paediatric model. We started from mathematical models previously validated and published by our group to have a solid basis from the mathematical point of view. Beside, we used both literature and data acquisition to collect all the parameters we need to switch from an adult to a paediatric model, by a proper tuning of the parameters of the mathematical equations. The adjusted hydraulic map of the vascular system is depicted in Fig. 2b.

## Results

### Arterial adjustments

**Fig. 3:**
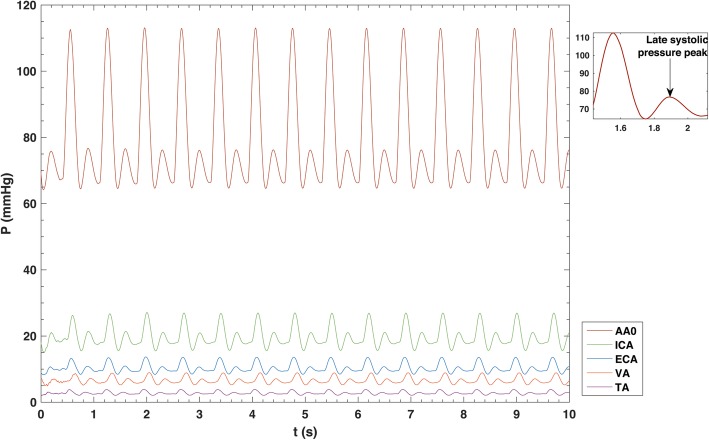
Cardiac and arterial pressure waveforms. The inset shows the zoom of one cardiac cycle. *AAo* Ascending aorta, *ECA* Extracranial artery, *ICA* Intracranial artery, *FA* Facial artery, *VA* Vertebral artery

The pressure pulse wave of Fig. 1, together with the contribution of the respiratory pulse are the input to the model. As shown in Fig. 3, the simulated pressure in the ascending aorta was 114/65 mmHg, in accordance with the paediatric cardiopulmonary care guide [27]. Figure 3 also reports the pressure pulse in the intracranial artery, extracranial artery, vertebral artery, and facial arteries calculated by the model.

The backward wave from ascending aorta to heart generate the late systolic or early diastolic pressure peak [18, 24]. This late pulse can be seen in Fig. 3. Moreover, the blood flows from the heart to the vessels and is divided among many branches. This must correspond to a change in the venous pressure. We can see from Fig. 3 that the model properly simulates both decrease in pressure amplitudes and mean values in the following order: ascending aorta > intracranial artery > extracranial artery > facial arteries [13, 16, 24].

**Fig. 4:**
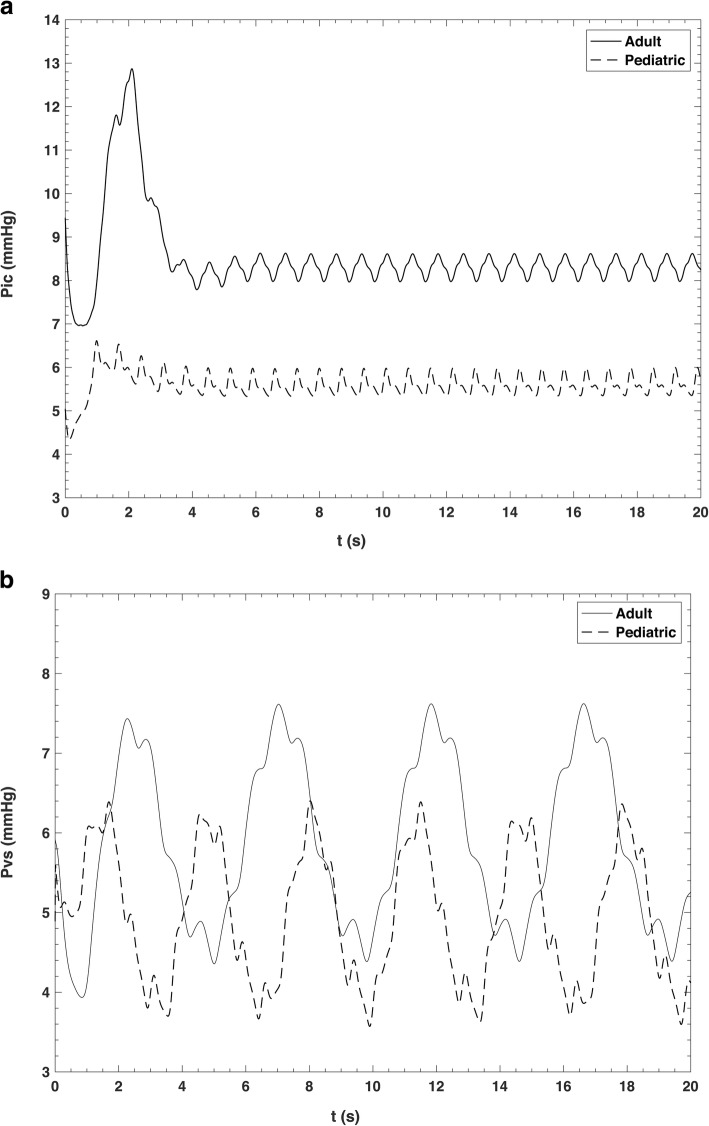
Comparison between (**a**) intracranial (*P*_ic_) and (**b**) venous sinus (*P*_vs_) pulse in paediatric (dashed line) and adult (solid line)

Figure 4 compares the simulated intracranial artery pressure and venous sinus pressure in paediatric subjects and adults. We see that the mean pressure values were less in lower age. Indeed, the mean values of intracranial artery pressure are 5.6 ± 0.2 (mean ± SD) and 8.3 ± 0.8 mmHg (mean ± SD), while the mean values of venous sinus pressure are 4.9 ± 0.6 and 5.9 ± 1.0 mmHg in paediatric subjects and adults, respectively. Moreover, we see that intracranial artery pressure was lower than venous sinus pressure. Hence, the simulated intracranial artery pressure and venous sinus pressure values were in agreement with the literature. The normal range of intracranial artery pressure was variable between 3 and 7 mmHg for paediatric subjects and less than 10−15 mmHg for adults [34, 35].

### Venous adjustments

**Fig. 5:**
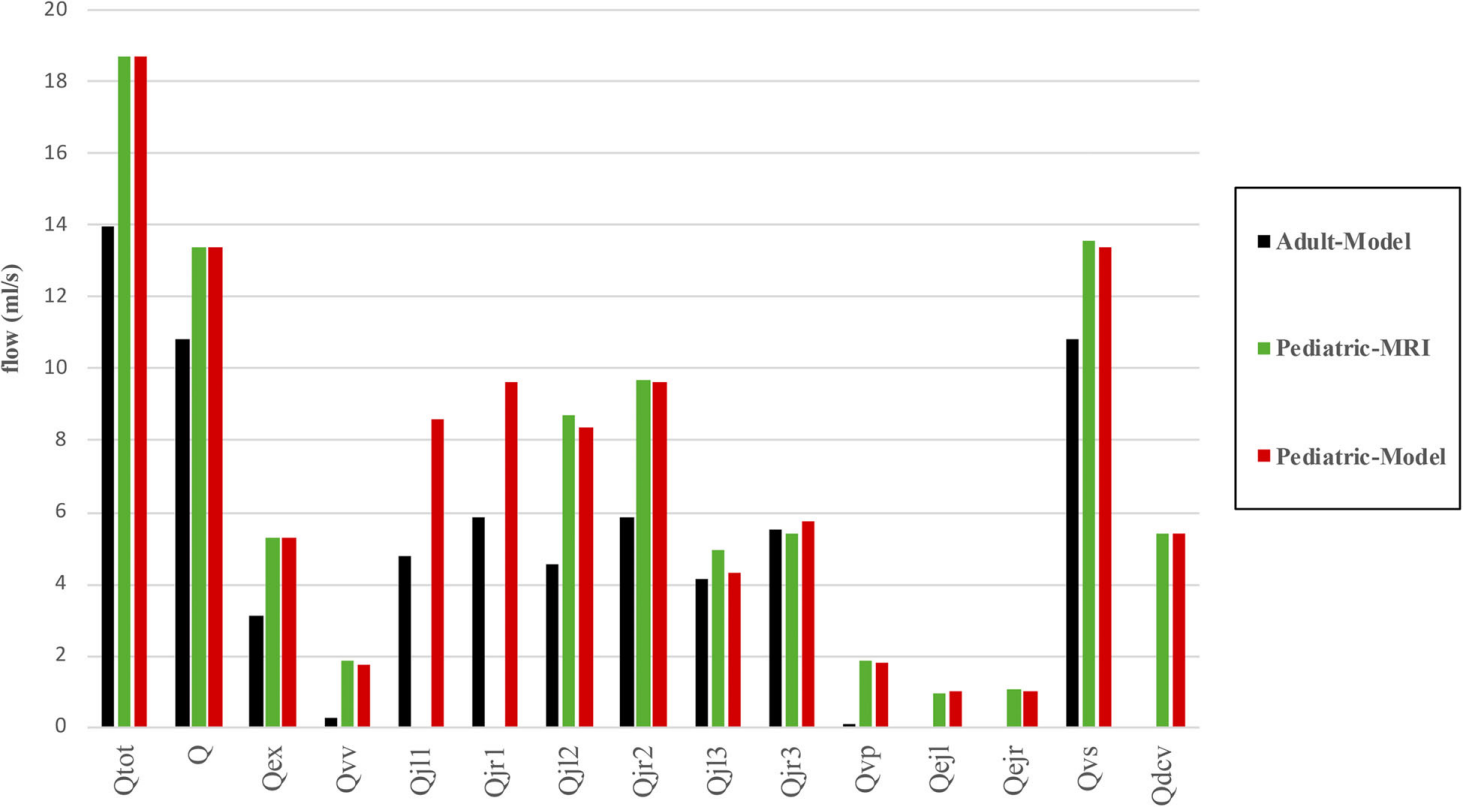
Comparison of simulated paediatric and adult flow rates, and paediatric experimental data. *Q* Cerebral blood flow, *dcv* Deep cervical vein, *ejl* Left external jugular vein, *ejr* Right external jugular vein, *Q*_*ex*_ Extracerebral blood flow, *jl1* Lower segment of the left jugular vein, *jl2* Middle segment of the left jugular vein, *jl3* Upper segment of the left jugular vein, *jr1* Lower segment of the right jugular vein, *jr2* Middle segment of the right jugular vein, *jr3* Upper segment of the right jugular vein, *Q*_*tot*_ Blood flow to the head, *vp* Venous plexus, *vs* Venous sinus, *vv* Vertebral vein

The venous part of the model was adjusted to simulate the main blood pathways from the brain. In the majority of individuals, IJVs are the main pathway and the flow at each segment (J1, J2, and J3) is more than that of vertebral veins or internal venous plexus flow [14, 34]. In this work, we refer to the flow rate in VV and DCV as summation of the left and right veins due to the low value of them. The intracranial flow rate is calculated by the summation of ICA left and right and basilar arteries [2]. Moreover, total blood flow is the flow rate at the level of neck arteries that supplies the intracranial and extracranial subsystems [14, 21]. Figure 5 shows the amount of the simulated flow rates at rest and makes a comparison between paediatric and adult flow rates.

There was good agreement between model response and paediatric MRI flow data. Moreover, flow rates were higher in paediatric rather than adult subjects, as expected.

**Fig. 6:**
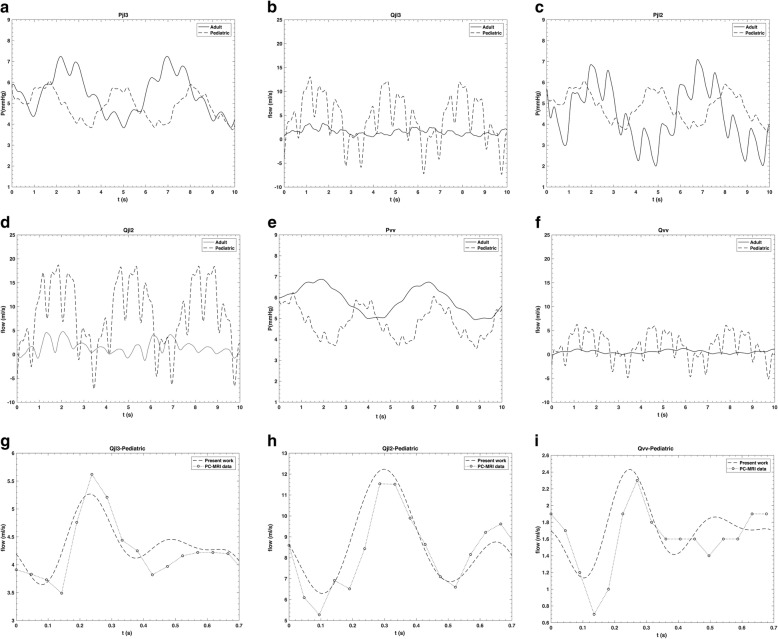
Comparison of the simulated pressure and flow rate at the level of IJV and VV for paediatric (dashed line), adult (solid line), and PC-MRI data (dotted line). *jl2* Middle segment of the left jugular vein, *jl3* Upper segment of the left jugular vein, *vv* Vertebral vein

Figure 6a–f shows how the model simulates the pressure and flow rate both in paediatric (dashed line) and adult (solid line) setting in IJV and vertebral veins. Figure 6g–i compares the simulated flow waveforms in IJV and VV against PC-MRI data. In this figure, the simulation of respiratory rate and so the effects on modeled flow rate was eliminated in order to make a comparison with phase-contrast data of one cardiac cycle. In order to quantify the agreement shown in Fig. 6g–i, we calculated the time-averaged difference between simulated (dashed line) and measured (dotted line) flow rates, during the reported cardiac cycle, for left J3, left J2, and vertebral vein. Such time-averaged difference was 1.1 ± 0.3 mL/s (mean ± SD), 3.7 ± 0.1 mL/s (mean ± SD), and 0.3 ± 0.2 mL/s (mean ± SD), respectively.

## Discussion

Based on a well-developed mathematical model for the simulation of blood circulation in adult humans [11], the present model has been tuned with respect to paediatric physiological parameters (average age 12 years, supine position).

In 2017, Cattermole et al. [35] reported a measured values of ascending aorta pressure of 80.7 mmHg (range 65.3 to 103.7 mmHg) in the same age range as this study (see Fig. 3). Our simulated pressure in the intracranial artery (22 ± 4 mmHg, mean ± SD, age range 2 to 18 years old) follows the trend of the data reported in literature (age range 20 to 60 years old and older), where blood pressure in intracranial artery increases with age. Indeed, on 2006 Hirata et al. [36] investigated blood velocity and pressure in intracranial artery and reported a mean value of 29.3 ± 6.4 mmHg (mean ± SD) for the age range from 20 to 40 years, 35.9 ± 8.0 mmHg (mean ± SD) from 40 to 60 years and higher values for higher ages.

The CBV increases rapidly from early childhood to preschool age up to 6 years, and then continuously decreases till adulthood [2, 8]. Moses et al. [8] reported a normalised different value of 10.6 ± 5.2 mL/100 mL/min between the mean age of 12 years and adults (mean age of 22 years) [8]. Wu et al. [2] reported a blood flow reduction rate of about 48% from the age of 12 to 40 years [2]. According to Fig. 5, the average inflow toward head and brain are 18.7 mL/s and 13.4 mL/s that is a 34% and 24% augmentation in paediatric rather than adult subjects. These values are within the range of previous reports [2, 5, 8].

Additionally, we can see from Fig. 5 the asymmetric behaviour of the cerebral venous drainage. Indeed, the right side flow rate is about 10% more than the left, as reported by the literature [14, 33, 34, 37].

The mean value of central venous pressure and EJV pressure in adults were studied by Leonard et al. [37]. They stated that mean EJV pressure is slightly higher than IJV pressure. They measured 8.3 ± 2.6 mmHg (mean ± SD) for IJV and 8.6 ± 2.8 mmHg (mean ± SD) for EJV in adults with a mean age of 66 years. As depicted in Fig. 6, mean IJV pressure in all three sections and vertebral vein pressure were 4.7 ± 0.8 mmHg (mean ± SD) and 4.8 ± 0.7 mmHg (mean ± SD) in paediatric subjects, and 5.6 ± 1.1 mmHg (mean ± SD) and 5.8 ± 0.7 mmHg (mean ± SD) in adults, respectively, showing that the model results are quite satisfactory. However, there is the need of more paediatric measurement at the IJV level and peripheral veins in order to produce a model with more details.

Results reported in Fig. 6a–f were satisfactory because the simulated pressure values were higher in adult than paediatric subjects, while flow rates were lower [2, 8].

Figure 6g–i represents a comparison between the simulated and measured flow rates in J3, J2, and vertebral vein as an example of the accuracy of our paediatric model. The correlation value between the simulated and PC-MRI data was 88%. The positive value of all the calculated time-averaged differences between simulated and measured flow rates means that, in the investigated anatomical regions, the mathematical model slightly overestimates the flow rate. The low SDs are index of good agreement between simulated and measured flow rates. As previously reported in literature [38], there is interest in properly simulate flow rate and pressure waveforms to discriminate between physiologic and pathologic subjects. The present model is able to simulate such waveforms for a paediatric setting.

Our model is a suitable computational tool to study the paediatric and adult haemodynamics. In a previous paper, Gadda et al. showed the ability of the model we used to simulate healthy and pathological cases [11, 14, 21]. Here, the model is expanded and adjusted to a paediatric setting by keeping the previous abilities. Therefore, following the pathway of Gadda et al. [14], a further step will be to collect MRI datasets from paediatric subjects with different kind of vascular diseases (*i.e.*, vessel occlusions at different levels of the vascular tree), and tune the paediatric model to properly simulate blood flows and pressure in those new pathological settings. For our work, the phase-contrast technique is important to assess the contribution of peripheral vessels in the human brain drainage. Indeed, PC-MRI is able to show that the external jugular, deep cervical and epidural veins play a crucial role. From this evidence, we decided to extend the anatomic map of the model from the work by Gadda et al. [11] to the version presented here. Moreover, in future work, we want to take advantage of our data from Sylvian aqueduct to improve the cerebrospinal fluid circulation related parameters and brain part of the model.

Further discussions are needed to assess the opportunity to use other MRI techniques for these purposes. We are currently going to use four-dimensional flow sequences. Such update will allow new measurements to further refine and validate the model. However, four-dimensional flow protocols are highly time-consuming to study blood flows at the neck level because of the volume involved. This protocol will extend the examination time by about 20 min, probably not acceptable for the paediatric tolerance and the review board.

Our study has limitations. To retrieve flow measurements from phase contrast imaging is sometimes not easy in small vessels, and even more difficult in veins due to the lower yields of the parameters in paediatric subjects. The patient selection protocol was affected by limitations that decrease the number of cases. Subjects were chosen among patients with brain MRI prescription. We considered two conditions to select a patient for the study: normal neck and brain blood circulation, and no sedation during MRI due to the influence of the anaesthesia medication on the brain flows [39].

According to Doepp et al. [15] there are two main categories of human blood drainage: jugular drainage (75% of the population) and non-jugular drainage (25% of the population). Our dataset is in perfect agreement, showing a subset of jugular drainers equal to 74% (26% non-jugular). Nevertheless, we decided to not separate the two subgroups in this study, focusing on the problem to tune an “adult based” model to paediatrics. Moreover, paediatric subjects are categorised in three subgroups: infants, preschool and old-children while there is no such a categorisation for adults. This implies a poor accuracy when we want to compare paediatric and adult subjects because the value of biofactors changes with age constantly. Insufficiency in the number of experimental studies and age categorisation may have affected the quality of numerical studies and modeling.

In conclusion, we modeled for the first time the paediatric haemodynamic circulation by using a parameter model consisting of a 0-D and 1-D algorithm. The previous well-described model [21] was adjusted in accordance to the differences between paediatric and adult subjects. The model was able to compute blood pressures and flow rates in accordance to the collected phase-contrast MRI data and literature. Moreover, it has the potential to simulate pathological conditions due to aging.

## Supplementary information

**Additional file 1.** Supplemental material to “paediatric haemodynamic modelling: development and experimental validation using quantitative flow MRI

## Data Availability

The datasets used and/or analysed during the current study are available from the corresponding author on reasonable request.

## Abbreviations

CBF: Cerebral blood flow
CBV: Cerebral blood volume
CO: Cardiac output
DBP: Diastolic blood pressure
DCV: Deep cervical vein
EJV: External jugular vein
HR: Heart rate
IJV: Internal jugular vein
J1: Internal jugular vein, segment 1
J2: Internal jugular vein, segment 2
J3: Internal jugular vein, segment 3
SBP: Systolic blood pressure
SD: Standard deviation
TBV: Total blood volume
