# Supplementary material for: Paediatric haemodynamic modelling: development and experimental validation using quantitative flow MRI
Source: Eur Radiol Exp. 2020 Mar 16;4:16. doi: 10.1186/s41747-020-0146-x (PMC7073351; doi:10.1186/s41747-020-0146-x)
Supplement: Supplementary file 1 — Additional file 1. Supplemental material to “paediatric haemodynamic modelling: development and experimental validation using quantitative flow MRI [file 41747_2020_146_MOESM1_ESM.docx]

**Supplemental material to “Paediatric hemodynamic modelling: development and experimental validation using quantitative flow MRI” – Appendix 1**

The 2D-PC-MR Images acquired from one of the volunteers.


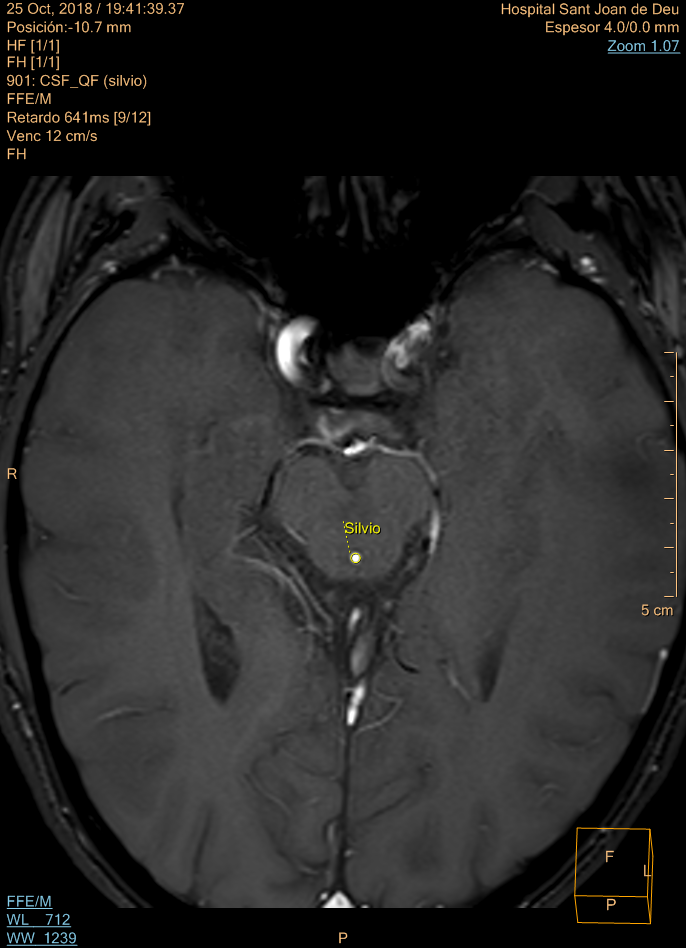

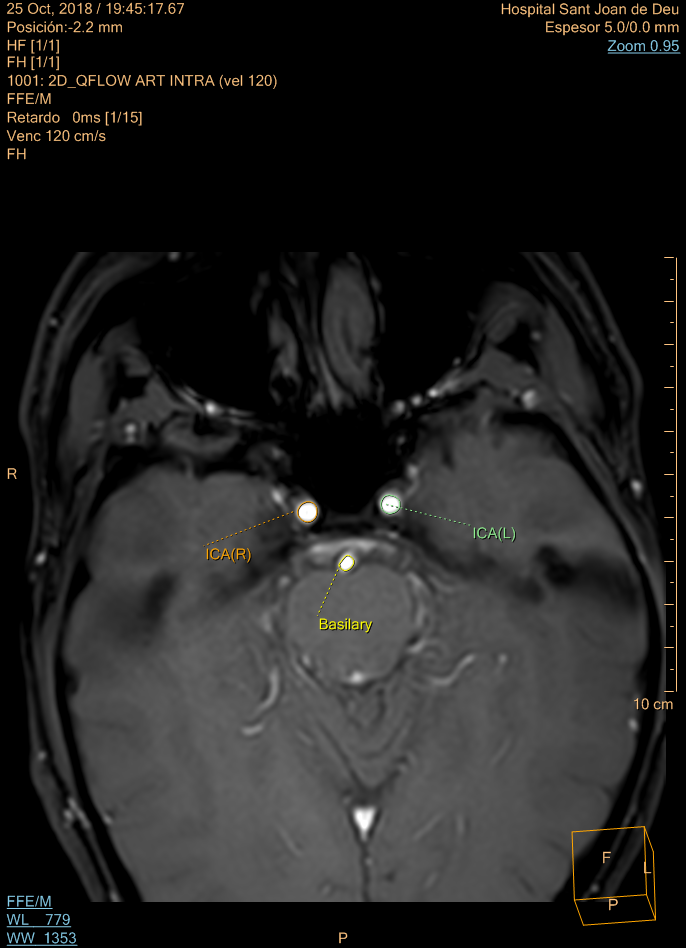

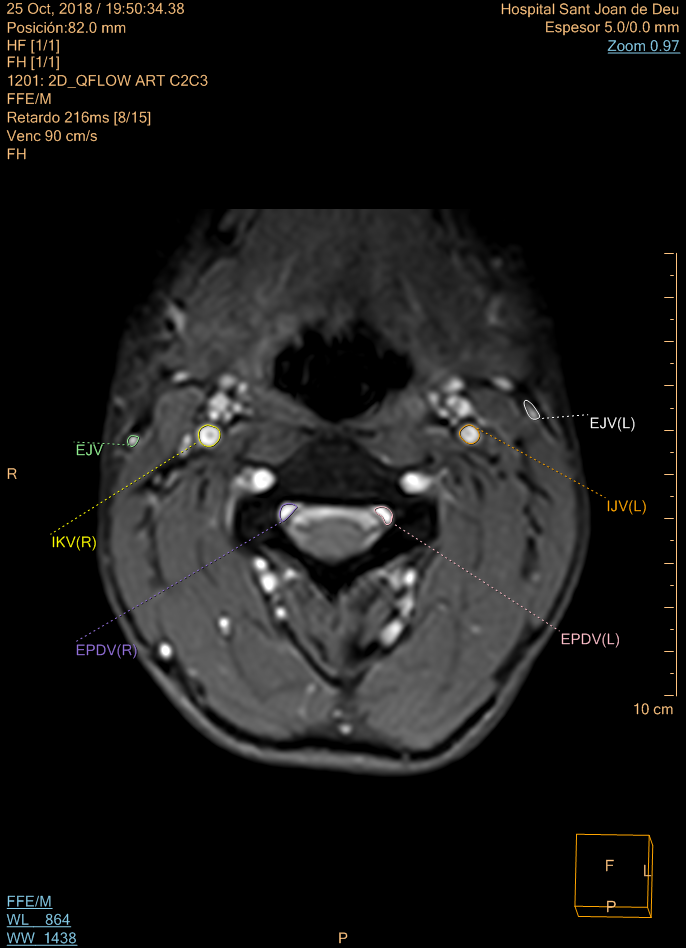

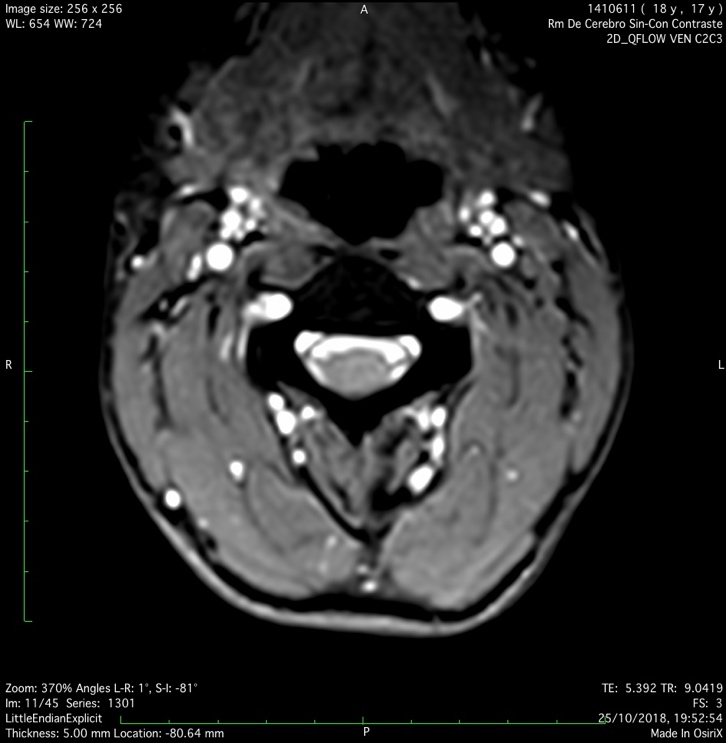

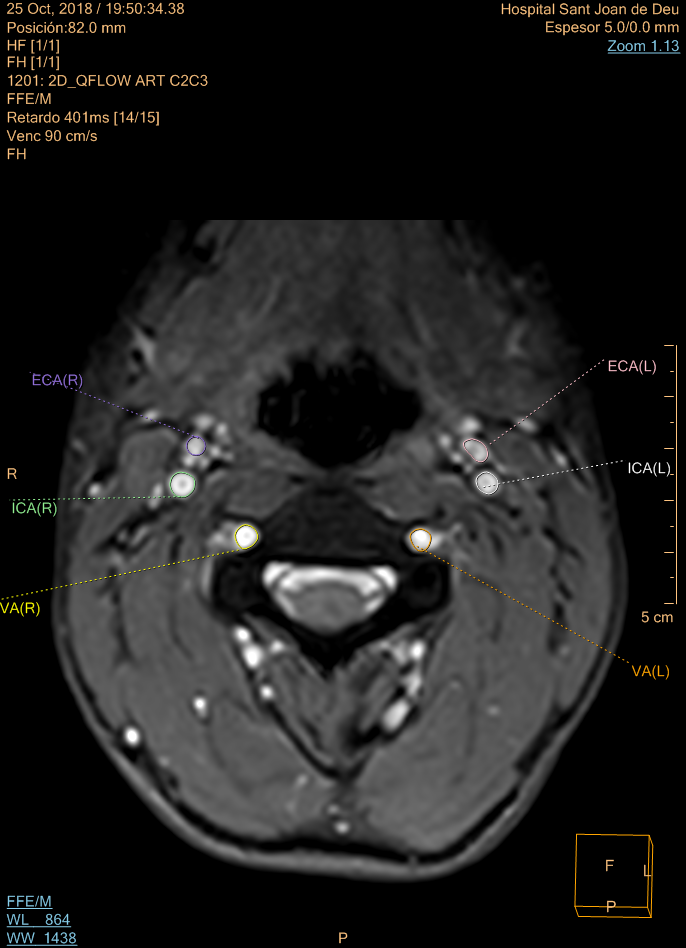

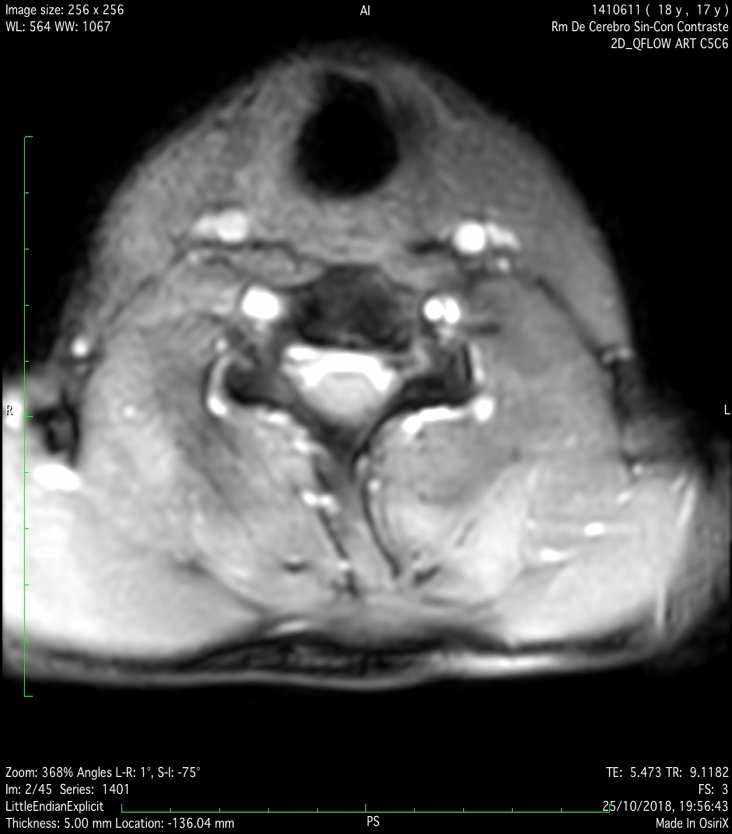


ART-C5/C6

VEIN-C5/C6

VEIN-C2/C3

ART-C2/C3

ART-BRAIN

CSF-SILVIO

**Supplemental material to “Paediatric hemodynamic modelling: development and experimental validation using quantitative flow MRI” – Appendix 2**

***Mathematical modelling***

The model consists of two main parts: a 0-D and a 1-D algorithm [1]. In the 0-D algorithm, the intracranial autoregulation mechanisms and cerebral outflow were modelled according to the model of Ursino et al [2,3]. According to the equations of the model, each vessel is considered as an electric element with a given value of resistance (R), capacitance (C) and conductance (G) of the blood vessel [3,4].

$R=\frac{8\eta L}{\pi r^{4}}$ (1)

$G=\frac{1}{R}$ (2)

$C=\frac{increase in volume (\Delta V)}{increase in pressure (\Delta P)}$ (3)

In the equations, the constant value ρ = 1060 kg/m^3^ , η = 3.8 × 10^-3^ Pa·s , g = 9.81 m/s^2^, L and r are the blood density, viscosity, gravity acceleration, length and radius of the vein, respectively.

To account for mass preservation in the whole 0-D model, the pressure change over time (dP/dt) is calculated according to the Kirchhoff law for each vessel of the whole network. For instance, the variation of pressure is calculated in the right J3 segment (jr3) by Equation 4 and 5 in supine position as fully explained in the papers by Gadda et al [5,6].

$\frac{dP}{dt}=\frac{1}{C_{jr3}}[\left( P_{vs}-P_{jr3} \right)G_{jr3}-\left( P_{jr3}-P_{c3} \right)G_{cjr3}-\left( P_{jr3}-P_{jr2} \right)G_{jr2}]$ (4)

$G_{jr3}=k_{jr3}\left[ 1+\left( \frac{2}{\pi} \right)\arctan\left( \frac{P_{vs}}{A} \right) \right]^{2}$ (5)

G has a switch-like function to simulate the collapsibility behaviour of the IJV.

In the 1-D algorithm, the whole body arterial circulation of the 55 main arteries is simulated by Majka et al [7]. Such model considers the cardiac pulse propagation through the vessels. Vessel walls and blood are modelled by using Navier-Stokes equation, in which blood was considered as a non-compressive Newtonian fluid. The flow rate, blood pressure change, and mean lumen area can be calculated as a function of time (t) and space (x) by the equations below [7,8,9,10]:

$Q(x,t)=A\times\bar{V}(x,t)$ (6)

$\frac{\partial Q}{\partial t}+\frac{\partial}{\partial x}(\frac{Q^{2}}{A})+\frac{A}{\rho}\frac{\partial P}{\partial x}=\frac{f}{\rho}$ (7)

$\frac{\partial A}{\partial t}+\frac{\partial Q}{\partial z}=0$ (8)

$P=P_{0}+\frac{E\pi R_{0}h}{A}(\sqrt{\frac{A}{A_{0}}}-1)+\frac{k\pi R_{0}h}{A}\frac{1}{2\sqrt{AA_{0}}}\frac{\partial A}{\partial t}$ (9)

where A is the internal area of the vessel at a given section, and *f* (*x*, *t*) is the frictional force per unit length. Parameters E and k are the effective Young modulus and elastic component of the vessel wall (subscript 0 refers to reference values). These equations satisfy the momentum and mass conservation laws, and describe the pressure-area relationship.

At any artery bifurcation, a parent artery (a) and two branched daughter arteries (d1, d2) are defined. The distribution of incoming fluid between daughters depends on the reflection and transmission coefficients, that can be calculated from the mechanical characteristics of the vessels such as impedance (Z), as described by equations 10 and 11 [7,9].

$Z=\frac{\rho c}{A_{0}}$ (10)

$R_{f}^{a}=\frac{\left( Z^{a} \right)^{-1}-\left( Z^{d1} \right)^{-1}-\left( Z^{d2} \right)^{-1}}{\left( Z^{a} \right)^{-1}+\left( Z^{d1} \right)^{-1}+\left( Z^{d2} \right)^{-1}}$ (11)

where c is the mean velocity of blood at the parent artery.

The model also takes into account the effects of breathing and carbon dioxide pressure, and simulates body posture change, aortic valve diseases stenosis, regurgitation, and the effect of exercise on cardiac response [1,5,7,9].

**Reference list**

1. Gadda G, Majka M, Zieliński P, Gambaccini M, Taibi A (2018) A multiscale model for the simulation of cerebral and extracerebral blood flows and pressures in humans. Eur J Appl Physiol 118(11):2443–2454. doi:10.1007/s00421-018-3971-3
2. Ursino M, Lodi CA (1997) A simple mathematical model of the interaction between intracranial pressure and cerebral hemodynamics. J Appl Physiol 82(4):1256–1269. doi:10.1152/jappl.1997.82.4.1256
3. Ursino M, Ter Minassian A, Lodi CA, Beydon L (2000) Cerebral hemodynamics during arterial and CO(2) pressure changes: in vivo prediction by a mathematical model. Am J Physiol Heart Circ Physiol 279(5):H2439–H2455. doi:10.1152/ajpheart.2000.279.5.H2439
4. Guyton AC (1991) The veins and their functions. In: Textbook of medical physiology, 8th edn. Saunders, Philadelphia
5. Gadda G, Taibi A, Sisini F et al (2016) Validation of a hemodynamic model for the study of the cerebral venous outflow system using MR imaging and echo‐color Doppler data. AJNR Am J Neuroradiol 37(11):2100–2109. doi:10.3174/ajnr.A4860
6. Gadda G, Taibi A, Sisini F, Gambaccini M, Zamboni P, Ursino M (2015) A new hemodynamic model for the study of cerebral venous outflow. Am J Physiol Heart Circ Physiol 308(3):H217– H231. doi:10.1152/ajpheart.00469.2014
7. Majka M, Gadda G, Taibi A, [Gałązka](https://www.ncbi.nlm.nih.gov/pubmed/?term=Ga%C5%82%C4%85zka%20M%5BAuthor%5D&cauthor=true&cauthor_uid=28415274) M, Zieliński P (2017) Earliest effects of sudden occlusions on pressure profiles in selected locations of the human systemic arterial system. Phys Rev E 95(3-1):032414. doi:10.1103/PhysRevE.95.032414
8. Qureshi MU, Vaughan GD, Sainsbury C et al (2014) Numerical simulation of blood flow and pressure drop in the pulmonary arterial and venous circulation. Biomech Model Mechanobiol 13(5):1137–1154. doi:10.1007/s10237-014-0563-y
9. Majka M, Gadda G, Taibi A, [Gałązka](https://www.ncbi.nlm.nih.gov/pubmed/?term=Ga%C5%82%C4%85zka%20M%5BAuthor%5D&cauthor=true&cauthor_uid=28415274) M, Zieliński P (2017) Protective properties of the arterial system against peripherally generated waves. Math Biosci 286:16–21. doi:10.1016/j.mbs.2017.01.007
10. Larrabide I, Blanco PJ, Urquiza SA et al (2012) HeMoLab – Hemodynamics Modelling Laboratory: an application for modelling the human cardiovascular system. Comput Biol Med 42(10):993–1004. doi:10.1016/j.compbiomed.2012.07.011
